# Ginkgo Biloba Extract and Long-Term Cognitive Decline: A 20-Year Follow-Up Population-Based Study

**DOI:** 10.1371/journal.pone.0052755

**Published:** 2013-01-11

**Authors:** Hélène Amieva, Céline Meillon, Catherine Helmer, Pascale Barberger-Gateau, Jean François Dartigues

**Affiliations:** 1 University of Bordeaux, ISPED, Centre INSERM U897-Epidemiologie-Biostatistique, Bordeaux, France; 2 University of Bordeaux, Department of Psychology, Bordeaux, France; University Of São Paulo, Brazil

## Abstract

**Background:**

Numerous studies have looked at the potential benefits of various nootropic drugs such as Ginkgo biloba extract (EGb761®; Tanakan®) and piracetam (Nootropyl®) on age-related cognitive decline often leading to inconclusive results due to small sample sizes or insufficient follow-up duration. The present study assesses the association between intake of EGb761® and cognitive function of elderly adults over a 20-year period.

**Methods and Findings:**

The data were gathered from the prospective community-based cohort study ‘Paquid’. Within the study sample of 3612 non-demented participants aged 65 and over at baseline, three groups were compared: 589 subjects reporting use of EGb761® at at least one of the ten assessment visits, 149 subjects reporting use of piracetam at one of the assessment visits and 2874 subjects not reporting use of either EGb761® or piracetam. Decline on MMSE, verbal fluency and visual memory over the 20-year follow-up was analysed with a multivariate mixed linear effects model. A significant difference in MMSE decline over the 20-year follow-up was observed in the EGb761® and piracetam treatment groups compared to the ‘neither treatment’ group. These effects were in opposite directions: the EGb761® group declined less rapidly than the ‘neither treatment’ group, whereas the piracetam group declined more rapidly (β = −0.6). Regarding verbal fluency and visual memory, no difference was observed between the EGb761® group and the ‘neither treatment’ group (respectively, β = 0.21 and β = −0.03), whereas the piracetam group declined more rapidly (respectively, β = −1.40 and β = −0.44). When comparing the EGb761® and piracetam groups directly, a different decline was observed for the three tests (respectively β = −1.07, β = −1.61 and β = −0.41).

**Conclusion:**

Cognitive decline in a non-demented elderly population was lower in subjects who reported using EGb761® than in those who did not. This effect may be a specific medication effect of EGb761®, since it was not observed for another nootropic medication, piracetam.

## Introduction

Age-related cognitive decline is one of the main challenges of mental health research. As no curative treatment for dementia presently exists, an alternative would be to find strategies that could contribute to attenuating cognitive decline in the elderly, which could in turn possibly delay the onset of dementia. A large number of interventional or observational studies have looked at the potential benefits of various pharmacological treatments. In particular, drugs controlling vascular factors such as statins have received much attention as a result of the growing emphasis on the vascular component of dementia and cognitive decline. However, these studies have yielded contrasting results [1]–[6]. As the neurodegenerative process is accompanied by exacerbated oxidative stress, anti-oxidant vitamins have also been considered as good candidates [7]. Even if the results of the few studies reporting potential benefits of vitamin E, beta-carotene or multi-vitamin supplements seem encouraging, overall the results are far from conclusive [8]–[11]. Certain studies have even raised safety concerns with doses of vitamins C or E far above the recommended dietary intake [12]–[15]. The meta-analysis published by Jia et al actually concluded that antioxidant supplements in elders aged over 65 years have no beneficial effects on cognitive decline [16]. This failure may be partly explained by inadequate amounts or types of antioxidants or inappropriate timing of the supplementation.

Drugs specifically prescribed for memory impairment such as nootropics and vasodilators represent obvious potential interventions to prevent cognitive decline. In France, *Ginkgo biloba* extract (EGb761®- Tanakan®) has been marketed for more than thirty years as a medication for memory impairment and is marketed in the United States as a dietary supplement. While the probably most well-known effect of *G. biloba* extract is the protection of neuronal cell membranes from free radical damage [17], the properties of EGb761® go beyond that simple antioxidant mechanism. It has been shown to reduce Aβ aggregation and toxicity [18], [19], participate to mitochondrial protection [20] and promote hippocampal neurogenesis [21]. EGb761® has been also shown to decrease blood viscosity and enhance microperfusion [22]. Several studies on rats models also showed that EGb 761® improves neurotransmission, in particular glutamatergic [23], dopaminergic and cholinergic system [24], [25]. Therefore, EGb761® can really be considered as a multi-target drug.

Recent reviews and meta-analyses of randomised controlled trials concluded that EGb761® is effective in the treatment of patients with dementia, including Alzheimer's disease, vascular dementia and mixed forms [26], [27], in particular in demented patients with neuropsychiatric symptoms [27]–[30]. Regarding prevention, only one observational study carried out in a cohort of elderly women has so far suggested the beneficial effect of vasodilators, including *G. biloba*, in delaying the onset of dementia [31].

However, two clinical trials, i.e. the GEM (for Ginkgo Evaluation of Memory) study conducted in 3069 participants aged 75 and over with mild cognitive impairment [32] and the GuidAge study conducted in 2854 participants aged 70 and over and reporting memory complaints [33] failed to confirm such results. In these studies, *G. biloba* at 120 mg twice a day was not effective in reducing the overall incidence of dementia or Alzheimer's disease. However, in both studies, as in another more limited feasibility trial [34], dementia was the main efficacy criteria and the follow-up period was relatively short (3.5 years in Dodge's study ; 6.1 years in the GEM study ; 5 years in the GuidAge study). Due to the particularly long pre-dementia phase of Alzheimer's disease, which is known to progress over decades, expecting a positive effect of *G. biloba* on the incidence of dementia over a period of 3 to 6 years would imply that *G. biloba* has a direct effect on the neurodegenerative process itself, which is probably an over-optimistic hypothesis. Another alternative interpretation of these negative results might be that *G. biloba* is no longer effective once the neurodegenerative process of dementia is too advanced. In this case, dementia outcome over a relatively short follow-up would not be the most relevant outcome to assess the efficacy of *G. biloba* on cognitive aging. Therefore, determining whether *G. biloba* is associated with long-term cognitive decline may be of interest in order to understand more clearly the usefulness of such treatment in the elderly.

This paper reports the effect of *G. biloba* on long-term cognitive decline within the PAQUID study. The PAQUID study is a large population-based study conducted in France, which has now 20 years of completed follow-up. As such, it is one of the largest and longest-running prospective studies of the natural history of cognitive decline and the incidence of dementia to have been performed. In this study, the rate of cognitive decline of elderly people reporting use of EGb761® was compared to that of participants reporting use of piracetam, another nootropic agent prescribed for memory impairment in subjects without dementia. Both groups were compared to those participants reporting use of neither of these drugs. The rate of cognitive decline was assessed over a period of 20 years during which cognition has been repeatedly assessed in a standardized manner with three common neuropsychological tests. Due to possible confounding effects of psychotropic drugs on cognitive decline, the association between EGb761® and consumption of psychotropic drugs, including antidepressants, benzodiazepines or antipsychotics, and its possible contribution to the results observed was also considered.

## Methods

### General study design

This was an exploratory retrospective analysis of longitudinal data collected prospectively over the twenty years of follow-up of the PAQUID cohort. The study population and methodology of the PAQUID cohort have been described in detail elsewhere [35]. Briefly, the study initially included a community based cohort of 3,777 elderly people, aged 65 and older, representative of Gironde and Dordogne, two areas in the southwest of France. The PAQUID Study was approved by the Ethics Committee of the Bordeaux University Hospital.

Data were collected by means of a questionnaire administered at home by trained psychologists at the time of inclusion and after 1, 3, 5, 8, 10, 13, 15, 17 and 20 years. Physical health was evaluated by self-reported diseases or symptoms (treated diabetes, a history of heart disease, stroke, or hypertension, and dyspnoea) and scales assessing functional status. Medication consumption was documented by self-report by participants at each visit. The questionnaire also included items about sociodemographic characteristics, objective and subjective physical health, functional assessment, depressive symptomatology, as well as the MMSE as an evaluation of global mental status [36]. In addition to the MMSE, two specific neuropsychological tests were proposed systematically at each visit. The multiple choice recognition form of the Benton Visual Retention Test (BVRT) was used to measure visual memory (scores range from 0 to 15) [37]. The Isaacs Set Test (IST) assessed verbal fluency by measuring the ability to generate lists of words in four semantic categories (colours, animals, fruits and cities) in a 30-second interval [38]. After the interview, the psychologists completed a standardised ancillary questionnaire designed to assign the DSM-III-R criteria for dementia [39]. Individuals who met criteria for dementia, as well as those presenting a decline of at least three points on the MMSE since the previous visit, were seen by a senior neurologist. The neurologist confirmed the dementia criteria and ascertained the NINCDS-ADRDA diagnostic criteria for Alzheimer's disease or the Hachinski score for vascular dementia [40], [41]. Additional paraclinical examinations could be performed if appropriate. All available information was reviewed by a panel of senior neurologists.

### Study sample

All 3777 participants of the PAQUID cohort were eligible for this analysis, with the exception of those with a diagnosis of dementia at the time of inclusion (n = 102) and those who reported taking both EGb761® and piracetam at any time of follow-up (n = 63). The 3612 eligible subjects were divided into three groups as follows: (1) subjects reporting use of EGb761® at any one of the ten assessment visits, (2) subjects reporting use of piracetam at any one of the ten assessment visits and (3) subjects not reporting use of either EGb761® or piracetam at any assessment visit.

### Statistical analysis

Baseline characteristics between the three treatment groups were compared using χ^2^ tests or analyses of variance as appropriate. The decline in score on the MMSE, IST and BVRT over the twenty year follow-up period was compared between the three treatment groups using linear mixed effect models [42]. This model offers the advantage of taking into account multiple determinations in an individual subject as well as the influence of potential confounding variables. The variable corresponding to use of EGb761® and piracetam was taken as a time-dependent variable. The output of the model was expressed as a β coefficient, which represents an effect size measure corresponding to the component of the change in score over the follow-up period that can be attributed to the treatment group. The statistical model controlled for the following confounding variables: age, gender, educational level (defined in two categories: no formal education and school certificate or higher), MMSE score at inclusion, depressive symptomatology measured with the Center for Epidemiological Studies Depression Scale (CES-D; cut-off score of 23 for women and 17 for men) and score on a memory complaints scale at inclusion [43], [44].

The association between EGb761® use and psychotropic drug consumption (antidepressants, benzodiazepines or antipsychotics) was assessed with a logistic regression model adjusted for the same confounding variables as those cited above. In order to assess the potential contribution of psychotropic drug consumption to the associations observed, the model was reiterated with additional adjustment for psychotropic drug consumption as a time-dependent variable.

Finally, a linear mixed effects model was applied to compare directly decline in cognitive scores between the EGb761® and piracetam treatment groups.

## Results

### Subjects

**Figure 1:**
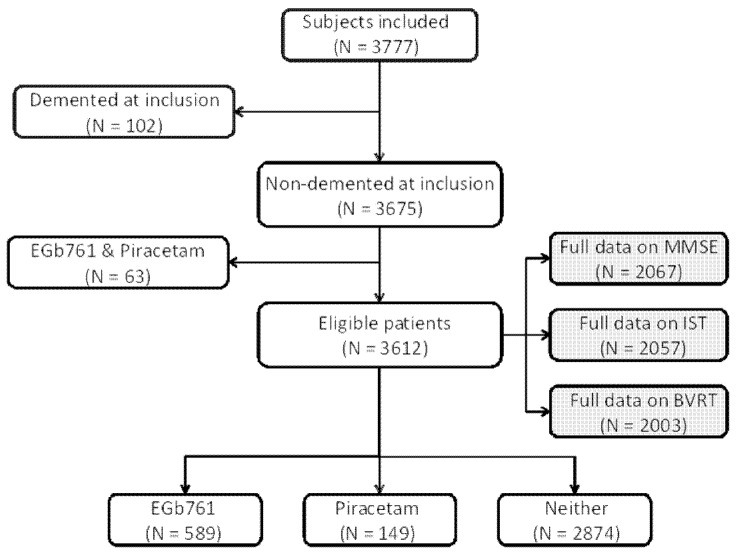
Selection of the study sample from the PAQUID cohort.

All 3777 participants in the PAQUID cohort were eligible for this analysis, with the exception of those with a diagnosis of dementia at the time of inclusion and those who reported taking both EGb761® and piracetam at any time. The study population consisted of 3612 subjects (95.6% of the total cohort). Of these, 589 (16.3%) reported use of EGb761® at any time of follow-up and 149 (4.1%) reported use of piracetam, whereas 2874 (79.6%) did not report use of either. For the analysis of decline in each cognitive test, the analysis was restricted to those subjects for whom data were available for the cognitive tests and for all relevant confounding variables that were to be included in the multivariate analysis. The subjects available for analysis corresponded to around two-thirds of the eligible population: 2003 for the BVRT, 2057 for the IST and 2067 for the MMSE. The composition of the study sample is illustrated in Figure 1.

The comparison of the characteristics of the three treatment groups at baseline is presented in Table 1. The three treatment groups did not differ in terms of age or number of medications, but significant differences were observed for all other variables. Subjects taking neither EGb761® nor piracetam tended to be more frequently men, less-educated, and to have less memory complaints than subjects taking either EGb761® or piracetam. Compared to subjects taking piracetam, subjects reporting EGb761® use were more frequently women and less frequently reported depressive symptoms or memory complaints. Baseline MMSE scores were slightly higher in the EGb761® group. At the end of follow-up, 73.3% of subjects in the EGb761® group, 86.6% in the piracetam group and 81.3% in the control group had died.

**Table 1:** Baseline characteristics of the three treatment groups.

Variable	EGb761® (n = 589)	Piracetam (n = 149)	Neither (n = 2874)	*p* (3-way)*	*p* (2-way)*
Age (years): mean (SD)	74.8 (6.6)	75.7 (6.6)	75.0 (6.9)	0.329	0.128
Gender (women): n (%)	435(73.9%)	91 (61.1%)	1556 (54.1%)	<0.0001	0.002
Education: n (%)				0.001	0.880
No formal education	172 (30.6%)	44 (31.2%)	1050 (38.4%)		
School certificate or higher	391 (69.4%)	97 (68.8%)	1685 (61.6%)		
Depressive symptoms: n (%)	60 (10.4%)	26 (17.9%)	388 (13.8%)	0.023	0.012
Baseline MMSE: mean (SD)	26.3 (2.9)	25.7 (3.9)	25.7 (3.5)	<0.001	0.040
Memory complaints: n (%)	283 (63.7%)	88 (75.2%)	984 (58.4%)	<0.001	0.020
Number of medications: mean (SD)	4.2 (2.7)	4.1 (2.7)	4.0 (2.8)	0.182	0.727

* Probability values are determined using the χ^2^ test or by analysis of variance as appropriate. The three-way determinations compared the distribution of variables between all three treatment groups and the two-way determinations between the EGb761® and piracetam groups only.

### Effects of treatment on cognitive decline

The Table 2 shows the means and standard deviations for the three cognitive scores at each time point. The analysis of decline in cognitive scores using the linear mixed effects model with repeated measures is displayed in Table 3. As can be seen, the MMSE score declined in the ‘neither treatment’ group by around 0.3 points between each study visit. A significant difference in the rate of change of MMSE score over the twenty-year follow-up period was observed in the EGb761® and piracetam treatment groups compared to the ‘neither treatment’ group. However, the direction of the treatment effect differed between the two treatments, subjects reporting use of EGb761® declining less rapidly than the ‘neither treatment’ group (p<0.0001), with a mean difference of 0.5 points on the MMSE per follow-up visit (around five points over the entire follow-up period). In contrast, the piracetam group declined more rapidly. With respect to the IST and the BVRT, no significant difference was observed between the EGb761® group compared to the ‘neither treatment’ group, whereas the piracetam group declined to a greater extent.

**Table 2:** Means and standard deviations for the three cognitive scores at each follow-up visit for the three treatment groups.

		Mean (SD)									
Test	Group	T0	T1	T3	T5	T8	T10	T13	T15	T17	T20
Mini Mental State Evaluation	Neither	25.7 (3.5)	26.7 (3.1)	26.2 (3.8)	26.1 (4.3)	25.7 (5.0)	24.6 (6.3)	24.4 (6.3)	24.1 (6.5)	24.0 (6.0)	24.0 (5.7)
	Piracetam	25.7 (3.9)	25.6 (4.6)	24.9 (5.2)	24.4 (6.3)	22.6 (8.4)	21.1 (9.2)	22.0 (7.7)	22.1 (7.6)	23.3 (5.7)	23.8 (6.5)
	EGb761®	26.3 (2.9)	27.1 (2.4)	26.7 (3.5)	26.5 (3.9)	26.1 (4.7)	25.4 (5.2)	24.3 (5.7)	24.3 (6.4)	23.5 (5.8)	23.7 (5.4)
Isaacs set test (30 sec)	Neither	34.4 (5.4)	35.2 (5.0)	34.9 (5.7)	39.2 (10.4)	38.3 (11.2)	37.0 (11.2)	37.7 (11.2)	37.4 (11.0)	36.2 (11.1)	34.6 (12.0)
	Piracetam	34.6 (5.6)	35.1 (5.5)	33.3 (6.6)	35.7 (11.3)	34.7 (11.2)	34.9 (11.3)	33.8 (13.1)	34.4 (10.0)	33.0 (11.2)	34.8 (12.2)
	EGb761®	35.3 (4.8)	35.7 (4.7)	35.7 (4.9)	39.2 (9.3)	37.8 (9.4)	37.2 (9.2)	36.2 (9.4)	35.9 (10.3)	33.5 (10.7)	31.4 (10.4)
Benton Visual Retention Test	Neither	10.1 (2.6)	10.9 (2.6)	10.8 (2.5)	10.9 (2.6)	10.5 (2.7)	10.6 (2.7)	10.6 (2.6)	10.7 (2.5)	10.4 (2.5)	10.6 (2.7)
	Piracetam	10.7 (2.4)	10.7 (2.8)	10.4 (2.8)	10.8 (2.5)	10.3 (3.2)	10.3 (2.7)	9.5 (3.1)	10.7 (2.4)	9.0 (3.4)	8.7 (3.3)
	EGb761®	10.3 (2.6)	10.9 (2.6)	11.0 (2.6)	11.1 (2.4)	10.9 (2.2)	10.6 (2.7)	10.5 (2.5)	10.5 (2.4)	10.2 (2.4)	10.2 (2.4)

**Table 3:** Comparison of change in cognitive outcomes over twenty years in the PAQUID cohort in subjects receiving EGb761® (n = 589) or piracetam (n = 149) compared to the ‘neither treatment’ group (n = 2874) (mixed linear effects model).

		Unadjusted for psychotropic drug use			Adjusted for psychotropic drug use		
Cognitive score	Variables	β^1^	SE	*p*	β^1^	SE	*p*
Mini Mental State Evaluation	Time	−0.315	0.013	<.0001	−0.302	0.013	<.0001
	Piracetam	−0.584	0.211	0.0057	−0.592	0.202	0.0034
	EGb761®	0.482	0.089	<.0001	0.461	0.085	<.0001
Isaacs Sets Test (30 sec)	Time	−0.290	0.020	<.0001	−0.258	0.019	<.0001
	Piracetam	−1.395	0.523	0.0077	−1.468	0.516	0.0045
	EGb761®	0.213	0.231	0.3561	0.271	0.227	0.2328
Benton Visual Retention Test	Time	−0.081	0.005	<.0001	−0.078	0.004	<.0001
	Piracetam	−0.438	0.194	0.0242	−0.470	0.184	0.0106
	EGb761®	−0.030	0.085	0.7223	−0.014	0.082	0.8631

1 Covariates: age, gender, educational level, MMSE score at inclusion, depressive symptomatology and memory complaints.

**Figure 2:**
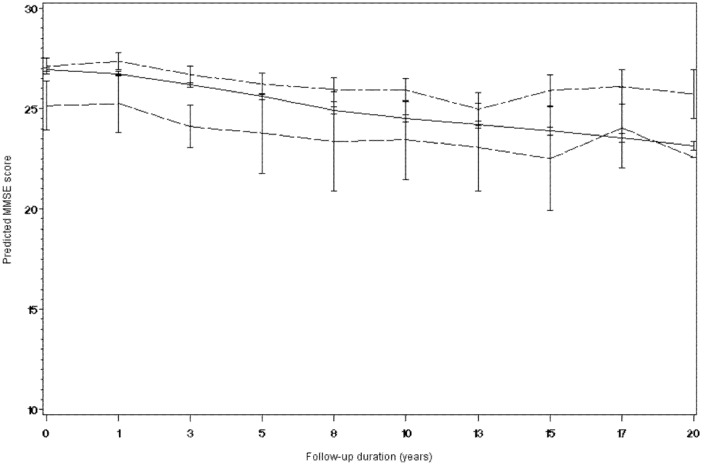
Estimated change in MMSE score over the twenty-year follow-up period in the three treatment groups. Legend: —— Neither treatment (n = 2874 at inclusion). – – – – EGb761® (n = 589 at inclusion). –·–·– Piracetam (n = 149 at inclusion).

A logistic regression model adjusted for the same confounding variables was performed to assess the association between EGb761® and psychotropic drug consumption (antidepressants, benzodiazepines or antipsychotics). The result showed that use of EGb761® was associated with significantly lower consumption of psychotropic drugs (OR 0.72, 95% Confidence Intervals: 0.57–0.91, *p* = 0.007). Due to the significant association between EGb761® use and reduced consumption of psychotropic drugs, the linear mixed effects model was reiterated adjusting for psychotropic drug consumption (Table 3). As can be seen, the beta coefficients remained unchanged, reflecting similar differences in cognitive decline between treatments groups after controlling for psychotropic drug use. The decline in MMSE score over time in the three treatment groups, as estimated by the model, is illustrated below in Figure 2.

In a second step, the linear mixed effects model was reiterated to compare the EGb761® and piracetam treatment groups directly (Table 4). As can be seen, the effect size on the MMSE corresponded to a less rapid decline by around one point on the MMSE per follow-up visit in the EGb761® group. In addition, a significant difference in rate of change was observed not only for the MMSE but also the other two tests of memory and verbal fluency. The findings remained essentially unchanged after controlling for psychotropic drug use (Table 4).

**Table 4:** Comparison of change in cognitive outcomes over twenty years in the PAQUID cohort between the EGb761® (n = 589) and piracetam treatment (n = 149) groups (mixed linear effects model).

		Unadjusted for psychotropic drug use			Adjusted for psychotropic drug use		
Cognitive score	Variables	β^1^	SE	p	β^1^	SE	p
Mini Mental State Evaluation	Time	−0.315	0.014	<.0001	−0.302	0.013	<.0001
	Piracetam vs EGb761®	−1.066	0.228	<.0001	−0.868	0.211	<.0001
Isaacs Sets Test (30 sec)	Time	−0.290	0.020	<.0001	−0.258	0.019	<.0001
	Piracetam vs EGb761®	−1.608	0.569	0.0047	−1.925	0.556	0.0005
Benton Visual Retention Test	Time	−0.081	0.005	<.0001	−0.078	0.004	<.0001
	Piracetam vs EGb761®	−0.407	0.211	0.0536	−0.573	0.202	0.0045

1 Covariates: age, gender, educational level, MMSE score at inclusion, depressive symptomatology and memory complaints.

## Discussion

This analysis of prospectively collected data on cognitive function over a twenty-year period has shown that the decline of the MMSE score in a population of non-demented subjects was lower in the group of subjects who reported using EGb761® at some time than in those who did not. The difference in MMSE score at the end of the follow-up period was around five points, which can be considered an important and clinically relevant difference. The predicted MMSE score at the end of the follow-up period remained above the threshold of 24 (roughly normal cognitive function) in the group using EGb761®, which is also of clinical relevance. This effect appears to be a specific medication effect of EGb761®, since it was not observed for another nootropic medication, piracetam, prescribed for the same condition as EGb761®, whose users performed less well all along the follow-up period in the three tests studied. The latter finding suggests that the observed beneficial effect of EGb761® on cognitive decline is not an artefact of greater motivation to preserve cognitive function which encourages subjects to seek medication in general for the management of memory complaints.

At first sight, these results may appear somewhat discordant with those trials such as the GEM [32] and the GuidAge [33] studies reporting no effect of the EGb761® on the risk of developing dementia, which led some authors to definitely conclude that ginkgo biloba is not effective for prevention of Alzheimer's disease [45]. However, our results may not be so inconsistent if one considers the following issues. Firstly, it is important to emphasise that these studies relied on volunteers presenting motivation to enter clinical trials testing drug candidates against memory decline. Such selection bias may have led to enroll participants particularly concerned about their memory problems for potentially different reasons. Supporting this issue is the particularly high rate of conversion to dementia in the GEM study where more than 17% of the participants developed dementia within the relatively short study follow-up, suggesting that a large proportion of participants were relatively advanced in the pre-clinical phase of dementia. The opposite seemed to occur in the GuidAge study conducted in this case in elderly people with memory complaints where the incidence of dementia was spectacularly low (actually less than half the expected value). This healthy participant effect has been noted in most dementia prevention trials and probably occurs because people who are more likely to volunteer for intervention trials might be already engaged in risk-reduction behaviors making them at less risk of dementia [46]. Secondly, as may be seen in many studies, including the present one, cognitive decline is a slow process in elderly non-demented subjects. For this reason, a short study follow-up may be insufficient to assess strategies, either pharmacological or non-pharmacological, that may have a significant but modest impact on cognitive decline. In our study, the treatment benefit associated with EGb761® only became clinically relevant after several years, a longer duration than that involved in the GEM study and the GuidAge study, the two clinical trials which reported no effect of EGb761® on the incidence of dementia. Another reason to believe that the possible effect of EGb761® may be appreciable in the long- rather that short-term relates to the long evolution of Alzheimer's disease before the dementia stage is attained. Dementia has been shown to be the end stage of a long evolutive process lasting more than a decade. Several long-term prospective studies have now clearly demonstrated differences on cognitive tests in individuals who ultimately developed dementia a decade or more prior to confirmed diagnosis, as well as steeper declines occurring three to four years prior to diagnosis [47]–[51]. These findings support the hypothesis that the pre-dementia phase is divided into distinct periods of decline, a first long period of slow cognitive decline and a shorter second period of faster decline, probably reflecting distinct pathogenetic steps. Such a hypothesis is consistent with the results of studies showing that the effects on cognitive decline of exposure to treatments such as non-steroidal anti-inflammatory drugs vary considerably according to the preclinical phase of Alzheimer's disease during which they are administered [52]. Therefore, regarding EGb761® exposure, expecting a protective effect on the rate of dementia incidence in a delay of 3 to 6 years before dementia diagnosis would imply a direct effect of EGb761® on the neurodegenerative process itself during the more aggressive period of the pre-clinical phase, which is probably an over-optimistic hypothesis for the mechanism of action of this drug. In addition, a previous analysis of the PAQUID study has shown that EGb761® may increase the probability of survival in the elderly population [53]. Since age is the strongest risk factor for developing dementia demonstrated to date, the increased risk of developing dementia due to longer survival could also offset any possible beneficial effects of EGb761® on cognitive function. Finally, one other reason could be that EGb761® has a symptomatic effect on cognition rather than a direct disease-modifying effect on the neurodegenerative process itself. Supporting this hypothesis are the quite numerous clinical data showing that Ginkgo biloba improves cognitive functioning in patients with Alzheimer's disease, with effect sizes similar to that obtained with other anti-dementia drugs such as acetylcholinesterase inhibitors [27].

An alternative explanation for the present findings showing lesser long-term cognitive decline in subjects reporting using EGb761® than in those reporting use of piracetam or neither drug could be related to differences in psychotropic use observed between the study groups. Indeed, our results showed that use of EGb761® was associated with significantly lower consumption of psychotropic drugs including antidepressants, benzodiazepines and antipsychotics. Given the well-characterised adverse effects of chronic psychotropic drug use on cognitive function [54]–[55], it was possible that the beneficial effect observed of EGb761® on cognitive decline was indirectly due to less psychotropic drugs consumption. However, when psychotropic drug use was taken into account as a possible confounding factor in the statistical model, the differences in the rate of cognitive decline between groups persisted, suggesting that the slower decline of MMSE scores in the EGb761® group cannot be explained by differences in psychotropic drug consumption.

Regarding, the finding of stronger decline in the group using piracetam, due to the small number of participants in this group, it is difficult to draw conclusions. The study of the relationship between cognitive decline and piracetam consumption was not the principal objective of our study, and was included in this study to have a point of comparison with a group of participants using a nootropic drug prescribed for the same condition as EGb761®. However, this result is somewhat intriguing and it would be important to replicate this result in another prospective study to draw reliable conclusions.

In conclusion, even though some points remain unclear, in particular the reason for the stronger decline observed in the piracetam group, or the question of a possible dose-effect of EGb761® that could not be presently tested, this study reports a beneficial effect of EGb761® on long-term cognitive decline as assessed by the MMSE in non-demented elderly people. This effect may be a specific medication effect of EGb761®, since it was not observed for another nootropic medication, piracetam, which was associated with more rapid decline in cognitive function, and cannot be accounted for by differences in psychotropic drug use.
